# URB597 protects against NLRP3 inflammasome activation by inhibiting autophagy dysfunction in a rat model of chronic cerebral hypoperfusion

**DOI:** 10.1186/s12974-019-1668-0

**Published:** 2019-12-09

**Authors:** Shao-Hua Su, Yi-Fang Wu, Qi Lin, Da-Peng Wang, Jian Hai

**Affiliations:** 1 Department of Neurosurgery, Tongji Hospital, Tongji University School of Medicine, 389 Xincun Road, Shanghai, 200065 China; 2 Department of Pharmacy, Institutes of Medical Sciences, Shanghai Jiao Tong University School of Medicine, Shanghai, 200025 China

**Keywords:** Chronic cerebral hypoperfusion, Autophagy, Mitophagy, NLRP3 inflammasome, ROS

## Abstract

**Background:**

Previous studies reported that URB597 (URB) had therapeutic potential for treating chronic cerebral hypoperfusion (CCH)-induced neuroinflammation and autophagy dysfunction. However, the interaction mechanisms underlying the CCH-induced abnormal excessive autophagy and neuroinflammation remain unknown. In this study, we investigated the roles of impaired autophagy in nucleotide-binding oligomerization domain-like receptor family pyrin domain-containing (NLRP) 3 inflammasome activation in the rat hippocampus and the underlying mechanisms under the condition of induced CCH as well as the effect of URB treatment.

**Methods:**

The CCH rat model was established by bilateral common carotid artery occlusion (BCCAo), and rats were randomly divided into 11 groups as follows: (1) sham-operated, (2) BCCAo; (3) BCCAo+autophagy inhibitor 3-methyladenine (3-MA), (4) BCCAo+lysosome inhibitor chloroquine (CQ), (5) BCCAo+microglial activation inhibitor minocycline, (6) BCCAo+ROS scavenger *N*-acetylcysteine (NAC), (7) BCCAo+URB, (8) BCCAo+URB+3-MA, (9) BCCAo+URB+CQ, (10) BCCAo+URB+minocycline, (11) BCCAo+URB+NAC. The cell localizations of LC3, p62, LAMP1, TOM20 and NLRP3 were assessed by immunofluorescence staining. The levels of autophagy-related proteins (LC3, p62, LAMP1, BNIP3 and parkin), NLRP3 inflammasome-related proteins (NLRP3, CASP1 and IL-1β), microglial marker (OX-42) and proinflammatory cytokines (iNOS and COX-2) were evaluated by western blotting, and proinflammatory cytokines (IL-1β and TNF-a) were determined by ELISA. Reactive oxygen species (ROS) were assessed by dihydroethidium staining. The mitochondrial ultrastructural changes were examined by electron microscopy.

**Results:**

CCH induced microglial overactivation and ROS accumulation, promoting the activation of the NLRP3 inflammasome and the release of IL-1β. Blocked autophagy and mitophagy flux enhanced the activation of the NLRP3-CASP1 inflammasome pathway. However, URB alleviated impaired autophagy and mitophagy by decreasing mitochondrial ROS and microglial overactivation as well as restoring lysosomal function, which would further inhibit the activation of the NLRP3-CASP1 inflammasome pathway.

**Conclusion:**

These findings extended previous studies indicating the function of URB in the mitigation of chronic ischemic injury of the brain.

## Introduction

A substantial amount of evidence indicates that chronic cerebral hypoperfusion (CCH), a state of chronic cerebral blood flow reduction, is associated with several cerebrovascular and neurodegenerative disorders including Alzheimer’s disease (AD), carotid stenosis/occlusion, cerebral arteriovenous malformation, dural arteriovenous fistula, moyamoya disease and cerebral small vessel disease [1–6]. In our previous study [7–11], we found that CCH may contribute to neuronal apoptosis, cognitive impairment, chronic neuroinflammatory responses and abnormal excessive autophagy in the frontal cortex and hippocampus of rats, resulting in cerebral ischemic damage. The fatty acid amide hydrolase inhibitor URB597 (URB) could be a candidate for alleviating chronic intracranial ischemic injury, as it could offer increased selective protection with less risk of the undesirable side effects that have been observed with cannabinoid receptor agonists capable of activating all accessible receptors indiscriminately [7–11]. Thus, we believe that URB might be a promising therapeutic agent for the treatment and management of CCH. However, the mechanisms underlying URB treatment are complex, and require further clarification.

Under conditions of cerebral ischemic insult, neuroinflammatory changes, such as the activation of microglia and astrocytes and the release of proinflammatory cytokines, initially serve as protective mechanisms. However, the unabated release of these proinflammatory cytokines subsequently interrupts the balance between proinflammatory and anti-inflammatory cytokines, contributing to neuronal damage. Chronic neuroinflammation is tightly associated with neuronal loss, neuronal cell death and accumulation of reactive oxygen species (ROS) [9, 12–14]. Nucleotide-binding oligomerization domain-like receptor family pyrin domain-containing (NLRP) 3 inflammasome is a member of the NLR family of innate immune cell sensors. It is a cytosolic multi-protein complex composed of NLRs, adaptor protein apoptosis-associated speck-like protein (ASC) and caspase-1 (CASP1) [15]. NLRP3 inflammasome is well-known to trigger the cleavage and activation of caspase-1, leading to maturation and secretion of interleukin-1β (IL-1β) and interleukin-18 (IL-18) [16]. Accumulating evidence indicates that overactivation of this inflammasome is critical for the pathogenesis of several disorders such as Crohn’s disease, atherosclerosis, gout and type 2 diabetes [17]. In the central nervous system (CNS), microglia are the principal neuroinflammatory cells. Constant microglial activation may promote the overactivation of the NLRP3 inflammasome, which mediates IL-1β-related inflammation [18]. Targeting the assembly and activity of the NLRP3 inflammasome would be a potential and novel therapy for cerebral ischemic stroke [19]. Hence, based on those facts, the regulation of NLRP3 inflammasome pathways in the presence of CCH is worth exploring.

Autophagy, an evolutionarily conserved cellular process, facilitates the continual delivery of damaged proteins and organelles to the lysosome for degradation, which is essential for cell survival and the maintenance of cellular homeostasis [20]. Autophagy may balance the beneficial and detrimental effects of neuroinflammation, and thereby may protect against chronic inflammatory neuronal injury. However, the interactions between the autophagy-lysosome pathway and neuroinflammation signaling are complex and controversial [21]. In some conditions, autophagy proteins could act as inducers and suppressors of inflammatory responses. In other conditions, inflammatory signals could, in turn, promote or inhibit the process of autophagy. To the best of our knowledge, under conditions of CCH, there is little data thus far that supports interactions between autophagy and neuroinflammation. Whether the autophagy-lysosome pathway participates in NLRP3 inflammasome activation has yet to be determined and understood. Therefore, elucidation of the possible mechanisms involved in CCH-induced abnormal excessive autophagy and NLRP3 inflammasome activation might provide new therapeutic targets for the treatment of CCH.

To clarify these issues, in the present study, we attempted to investigate the roles of impaired autophagy involved in NLRP3 inflammasome activation and the underlying mechanisms under conditions of CCH as well as treatment with URB.

## Materials and methods

### Animals

Male Sprague-Dawley rats, weighing 230–250 g, were obtained from the experimental animal center of Shanghai SIPPR-BK Laboratory Animal Co. Ltd. (Shanghai, China). Prior to the experiments, the rats were housed under standard conditions of temperature (23 ± 1 °C), humidity (60%) and light (12:12 h light/dark cycle, lights on from 08:30 to 20:30), with free access to food and water. After 1 week of acclimatization, the rats were randomly divided into 11 groups (five rats per group) as follows: (1) sham-operated, (2) bilateral common carotid artery occlusion (BCCAo), (3) BCCAo+autophagy inhibitor 3-methyladenine (3-MA; Sigma-Aldrich, St. Louis, MO, USA), (4) BCCAo+lysosome inhibitor chloroquine (CQ Sigma), (5) BCCAo+microglial activation inhibitor minocycline (Sigma-Aldrich), (6) BCCAo+ROS scavenger *N*-acetylcysteine (NAC; Sigma), (7) BCCAo+URB (Cayman Chemicals, Tallinn, Estonia), (8) BCCAo+URB+3-MA, (9) BCCAo+URB+CQ, (10) BCCAo+URB+minocycline, (11) BCCAo+URB+NAC. After 12 weeks, all rats were euthanized by deeply sedating them with 100% O^2^/5% isoflurane, followed by decapitation. Their brains were immediately removed for experiments or stored at − 70 °C for later use.

Experimental protocols were performed according to Chinese legislation on the use and care of laboratory animals and were approved by the Animal Laboratory Center of Tongji University School of Medicine.

### CCH model

The maximum chronic hypoperfusion phase in a rat model of CCH induced by BCCAo may persist for 12 weeks [22], which was the experimental interval used in this study. Rats were anesthetized with sodium pentobarbital [50 mg/kg, intraperitoneal (i.p.) injection]. A ventral midline incision was made, and both common carotid arteries were bilaterally separated from the carotid sheath and vagus nerve. Each common carotid artery was doubly ligated with a 5-0 silk suture approximately 8 to 10 mm inferior to the origin of the external carotid artery. Then, the incision was sutured. Sham-operated animals were subjected to the same procedure but without arterial ligation. After the surgical procedures, all rats received painkillers twice a day for 1 week.

### Drug administration

URB (0.3 mg/kg/day, i.p.), CQ (25 mg/kg/day, i.p.), minocycline (50 mg/kg/day, i.p.), NAC (20 mg/kg/day, i.p.) and 3-MA [3 μg/day, intracerebroventricular (i.c.v.) injection] dosages were selected based on previous studies [11, 23–25]. For baseline balance, all experimental rats received the intracerebroventricular catheter implantation. Afterwards, all rats received antibiotics injections for 1 week. Furthermore, WBC and CRP were detected for all rats once per week. Rats with significant inflammatory changes (pus from the operative incision, significantly higher WBC and CRP counts, etc.) were excluded from our study. All experimental drugs were dissolved in a solvent consisting of 99% sterile saline and 1% DMSO, while the sham-operated and BCCAo groups were daily administered an equivalent volume of vehicle solution (99% sterile saline and 1% DMSO). All experimental drugs were administered after BCCAo for 12 consecutive weeks.

Guide catheters were implanted into both cerebral ventricles (distances from bregma: anteroposterior, 0.5 mm; mediolateral, 1.0 mm; depth, 2.0 mm) [26]. Daily infusion of 3-MA was delivered using a microinjector (KD Scientific Inc., Holliston, MA, USA) into both cerebral ventricles at a rate of 2.5 μL/min for 12 weeks according to an earlier report [7–9, 11, 27] and protocols previously established in our laboratory.

### Electron microscopy

Briefly, according to our previous study [11], coronal slices in the CA1 hippocampal area were cut to a thickness of 1 mm and postfixed with 2.5% glutaraldehyde overnight at 4 °C. Then, these slices were washed with 0.1 mol/L PBS three times, and were postfixed in 1% osmium tetroxide for 2 h at 4 °C. Subsequently, the blocks were dehydrated in graded ethanol and embedded in epoxy resin. Randomly selected ultrathin sections (60–70 nm) were poststained with uranyl acetate and lead citrate and examined under an electron microscope (Philips, Amsterdam, Netherlands).

### Isolation of mitochondria

The mitochondrial fraction was isolated using a Qproteome Mitochondria Isolation Kit (Qiagen, Düsseldorf, Germany). Briefly, 20 mg of tissues were cut into pieces and homogenized in 2 mL lysis buffer with protease inhibitor solution. The supernatant (containing cytosolic proteins) was collected after centrifugation at 1000×*g* for 10 min at 4 °C. The pellet was resuspended and disrupted in 1.5 mL ice-cold disruption buffer. After centrifugation at 1000×*g* for 10 min at 4 °C, the supernatant was collected and centrifuged at 6000×*g* for 10 min. The pellet (containing mitochondria) was resuspended in 750 μL mitochondrial purification buffer and added to the top of a mitochondrial purification buffer layer (500 μL disruption buffer under 750 μL mitochondrial purification buffer). After centrifugation at 14,000×*g* for 15 min, a pellet or band containing mitochondria formed in the lower part of the tube and was transferred to a new tube. The suspension was washed three times with 1.5 mL mitochondrial storage buffer by centrifuging at 8000×*g* for 10 min. The highly purified mitochondria were resuspended in mitochondrial storage buffer. Fresh mitochondria were used for membrane potential detection. Mitochondrial and cytosolic fractions were stored at − 70 °C until further use.

### Western blot analysis

Samples (20 μg of protein) were separated by 8%, 10%, or 12% sodium dodecyl sulfate polyacrylamide gel and transferred to a nitrocellulose membrane. The membrane was blocked with 5% non-fat milk and 0.1% Tween-20 in Tris-buffered saline for 1 h and then incubated overnight at 4 °C with primary antibodies against the following proteins: microtubule-associated protein 1 light chain 3 (LC3, 1:200, Abgent), p62 (1:400, Abcam), lysosomal-associated membrane protein 1 (LAMP1, 1:1000, Cell Signaling Technology), parkin (1:1000, Cell Signaling Technology), BCL-2/adenovirus E1B (19 K)-interacting protein (BNIP3, 1:100, Cell Signaling Technology), NLRP3 (1:1000, Abcam), CASP1 (p20, 1:1000, Abcam), IL-1β (p17, 1:1000, Abcam), OX-42 (1:600, Santa Cruz Biotechnology), cyclooxygenase-2 (COX-2, 1:1500, Cell Signaling Technology), inducible nitric oxide synthase (iNOS, 1:1000, Cell Signaling Technology), voltage-dependent anion-selective channel 1 (VDAC1, 1:1000, Santa Cruz Biotechnology) and β-actin (1:5000, Abcam). The membrane was then incubated with the appropriate horseradish peroxidase-conjugated goat anti-rabbit or anti-mouse IgG secondary antibodies for 1 h at room temperature. Protein bands were detected by applying SuperSignal-enhanced chemiluminescent substrate solution (Millipore, Watford, UK). The proteins were quantified by OD ratio using β-actin as the control. In the case of mitochondria, VDAC1 was employed as the loading control.

### Immunofluorescence staining

The hippocampus was dissected from the brain and then was serially dehydrated, embedded in paraffin and cut into 5-μm thick coronal sections for immunofluorescence. Briefly, paraffin-embedded sections were deparaffinized and washed three times with PBS for 5 min. Then, the sections were immersed in EDTA-Tris solution (pH 9.0) for 30 min at 98 °C for antigen retrieval and rinsed three times with PBS for 5 min. Subsequently, the slides were incubated with 10% non-immune goat serum for 30 min at room temperature to block non-specific staining. After that, the slides were incubated in humidified chambers at 4 °C overnight with primary antibodies as follows: LC3 (1:100, Abgent), p62 (1:100, Abcam), LAMP1 (1:200, Cell Signaling Technology), TOM20 (1:1000, Santa Cruz) and NLRP3 (1:500, Abcam). The next day, after washing these sections in PBS, tetramethylrhodamine isothiocyanate (TRITC)-conjugated anti-mouse/rabbit secondary antibodies (1:200, Santa Cruz Biotechnology) were applied for 1 h at 37 °C. Finally, all stained specimens were observed under a confocal laser scanning microscope (Leica, Wetzlar, Germany). For the quantitative analysis, the average score of five randomly selected areas was calculated using National Institutes of Health (NIH) Image Pro Plus 6.0 software.

### Enzyme-linked immunosorbent assay

The amounts of tumor necrosis factor (TNF)-α and IL-1β in the brain tissues were quantified using an enzyme-linked immunosorbent assay (ELISA) kit (R&D Systems, Minneapolis, MN, USA), according to the manufacturer’s instructions. Briefly, equal amounts of proteins were loaded into all wells, followed by measurements of optical density (OD) in a plate reader at a wavelength of 450 nm, with analyses for concentrations based on a standard curve. For convenience, all results were expressed as pg/mg protein.

### Dihydroethidium staining

Reactive oxygen species (ROS) were determined by dihydroethidium (DHE) staining as described previously [9, 28]. Briefly, the sections were incubated in 10 mmol/L DHE (Sigma-Aldrich, St. Louis, MO, USA) at room temperature for 30 min without light exposure. All stained slices were observed under a fluorescence microscope (Olympus IX71, Melville, NY, USA). For the quantitative analysis, the average score of five randomly selected areas was calculated using NIH Image Pro Plus 6.0 software.

### Statistical analysis

The data in our study are expressed as mean ± standard error of the mean (SEM). Statistical analyses were performed using one-way analysis of variance (ANOVA) with Dunnett’s post-hoc test. Values of *P* < 0.05 were used as the criterion for statistical significance.

## Results

### CCH-induced autophagy dysfunction may be correlated with IL-1β secretion in rat hippocampus

**Fig. 1:**
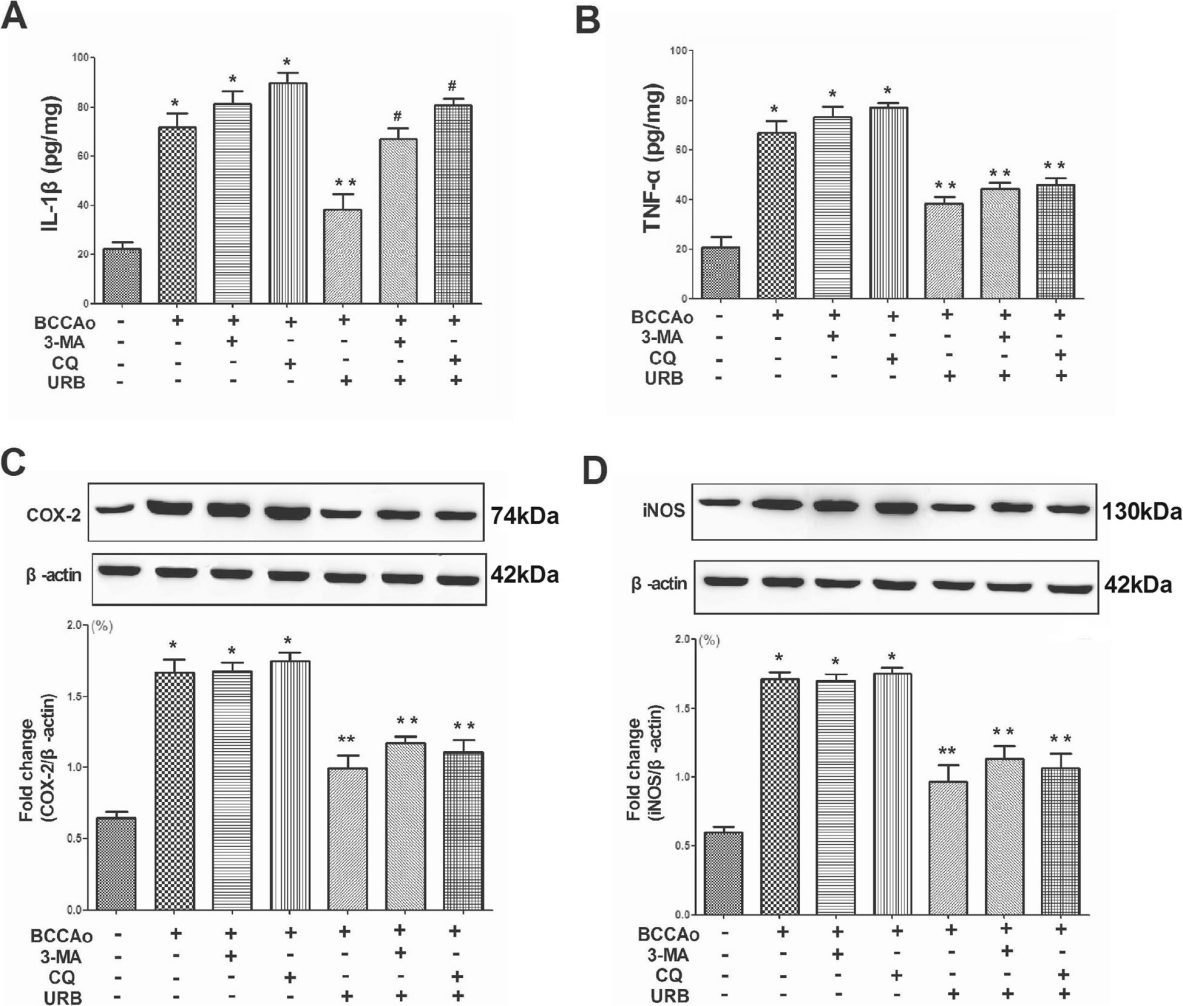
Effects of URB, autophagy inhibitor 3-MA and lysosome inhibitor CQ on CCH-induced up-regulation of proinflammatory cytokines. **a** The protein levels of IL-1β in the hippocampus. **b** The protein levels of TNF-α in the hippocampus. **c** Representative western blot and relative optical density analysis of COX-2 and β-actin in the hippocampus. **d** Representative western blot and relative optical density analysis of iNOS and β-actin in the hippocampus. Data are expressed as mean ± SEM (*n* = 5). *P < 0.05 versus sham group. **P < 0.05 versus BCCAo group. ^#^*P* < 0.05 versus BCCAo+URB group

In our previous studies [9, 11], we found that CCH caused the release of proinflammatory cytokines and induced lysosomal dysfunction, which may promote the accumulation of autophagosomes, resulting in abnormal excessive autophagy. Since it has been reported that autophagy dysfunction may contribute to the induction of neuroinflammation [29, 30], we examined whether there was a link between abnormal excessive autophagy and neuroinflammation in the presence of CCH. We investigated the expression of inflammatory markers, including TNF-α, IL-1β, COX-2 and iNOS, in the hippocampus. We found that CCH significantly increased TNF-α, IL-1β, COX-2 and iNOS levels in the rat hippocampus as compared with the sham group (Fig. 1a–d). However, treatment with URB significantly reversed this tendency. Interestingly, treatment with the autophagy inhibitor 3-MA and lysosome inhibitor CQ significantly neutralized the effects of URB and further promoted the expression of IL-1β after URB treatment (Fig. 1a). These results indicated that autophagy dysfunction may be involved in IL-1β secretion after CCH and URB treatment.

### URB inhibits CCH-induced NLRP3 inflammasome activation in rat hippocampus

**Fig. 2:**
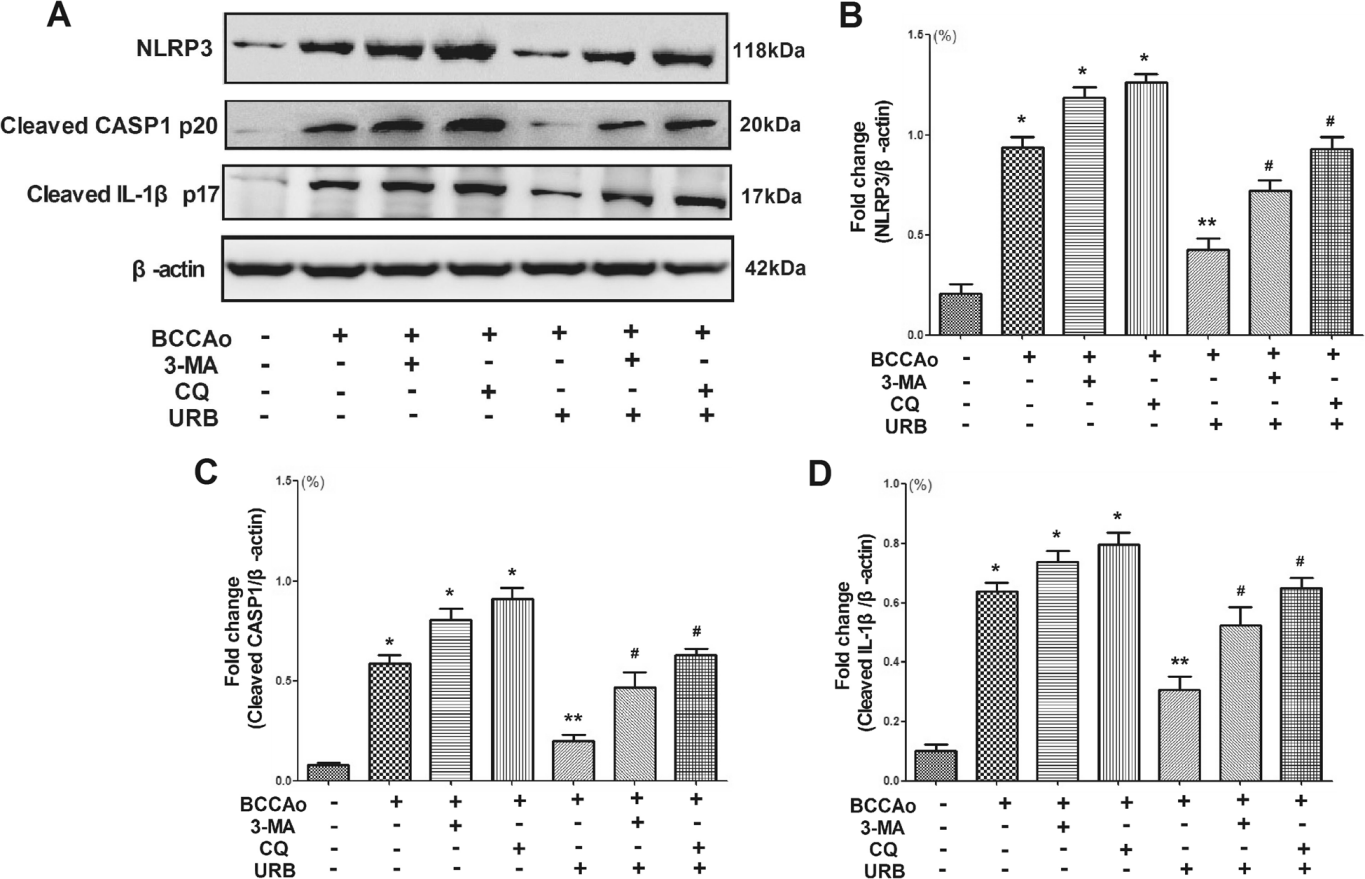
Effects of URB, autophagy inhibitor 3-MA and lysosome inhibitor CQ on CCH-induced NLRP3 inflammasome activation. **a** Representative western blot for NLRP3, cleaved CASP1, cleaved IL-1β and β-actin in the hippocampus. **b** Relative optical density analysis for NLRP3 in the hippocampus. **c** Relative optical density analysis for cleaved CASP1 in the hippocampus. **d** Relative optical density analysis for cleaved IL-1β in the hippocampus. Data are expressed as mean ± SEM (*n* = 5). *P < 0.05 versus sham group. **P < 0.05 versus BCCAo group. ^#^*P* < 0.05 versus BCCAo+URB group

The inflammasome is a cytosolic multi-protein complex composed of NLRs, adaptor protein ASC and CASP1 [15]. The NLRP3 inflammasome represents a primary pathway that triggers the activation of CASP1 and subsequently the maturation of IL-1β [31]. Considering that we found autophagy dysfunction may be involved in IL-1β secretion after CCH, the protein expressions of NLRP3, cleaved caspase-1 and cleaved IL-1β were analyzed in the hippocampus. Compared with the sham group, the expressions of NLRP3, cleaved caspase-1 and cleaved IL-1β were significantly increased by CCH, but this increase was significantly attenuated by URB treatment, suggesting that URB could suppress NLRP3-mediated CASP1 activation and mature IL-1β secretion. Moreover, the autophagy inhibitor 3-MA and lysosome inhibitor CQ notably neutralized the effects of URB and further increased NLRP3, cleaved caspase-1 and cleaved IL-1β levels after URB treatment (Fig. 2b–d). These results suggested that the autophagy-lysosome pathway might participate in CCH-induced activation of the NLRP3 inflammasome and subsequent release of IL-1β.

### URB alleviates CCH-induced NLRP3 inflammasome activation by promoting the restoration of lysosomal function

**Fig. 3:**
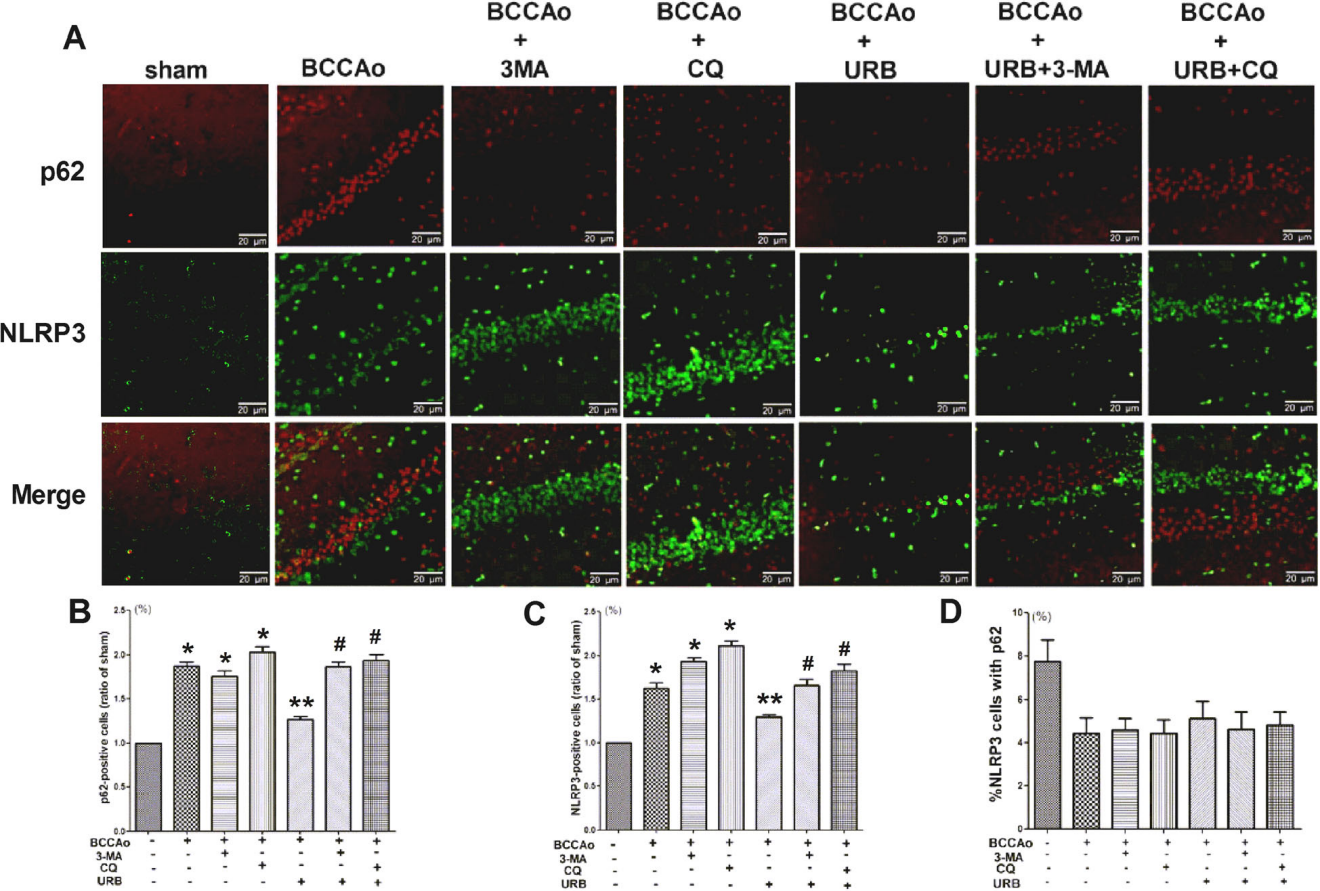
p62 may not be necessary for removing NLRP3 inflammasome in the process of autophagy after URB treatment. **a** Representative immunofluorescence staining for NLRP3 and p62 in the hippocampal CA1 region. (scale bars = 20 μm). *Green puncta*: NLRP3 inflammasome; *red puncta*: p62; *yellow puncta*: p62/NLRP3 overlap. **b** Relative level of p62 (ratio of the sham group). The p62-positive puncta in the sham group are set to 1. **c** Relative level of NLRP3 (ratio of the sham group). The NLRP3-positive inflammasome in the sham group is set to 1. **d** Relative level of p62/NLRP3 overlap (p62/NLRP3 overlap/mm^2^). Data are expressed as mean ± SEM (*n* = 5). *P < 0.05 versus sham group. **P < 0.05 versus BCCAo group. ^#^*P* < 0.05 versus BCCAo+URB group

**Fig. 4:**
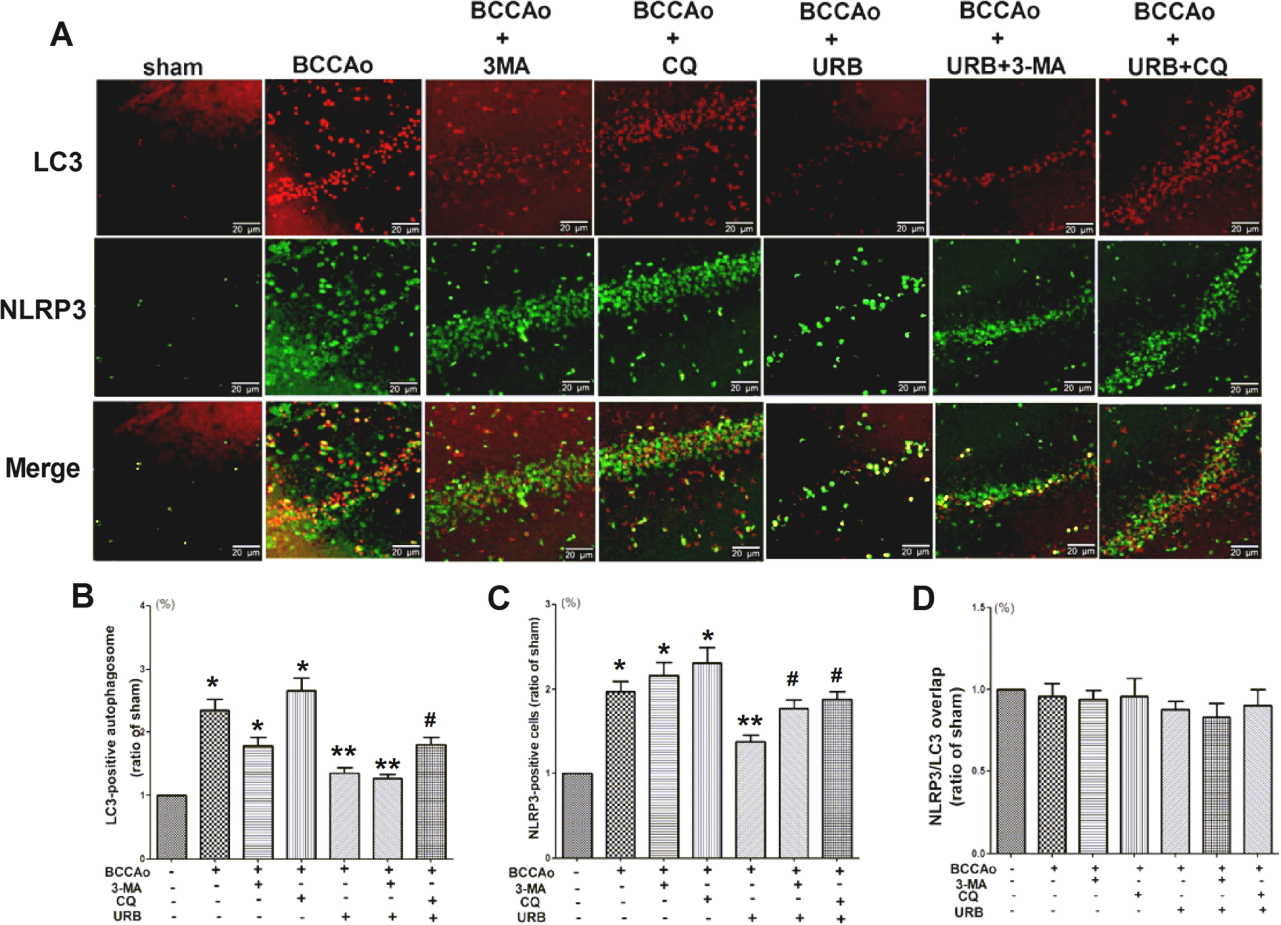
The activation of CCH-induced NLRP3 inflammasome may not be restrained by controlling the autophagosome formation stage after URB treatment. **a** Representative immunofluorescence staining for NLRP3 and LC3 in the hippocampal CA1 region. (scale bars = 20 μm). *Green puncta*: NLRP3 inflammasome; *red puncta*: LC3-positive autophagosome; *yellow puncta*: LC3/NLRP3 overlap. **b** Relative level of LC3 (ratio of the sham group). The LC3-positive autophagosome in the sham group is set to 1. **c** Relative level of NLRP3 (ratio of the sham group). The NLRP3-positive inflammasome in the sham group is set to 1. **d** Relative level of LC3/NLRP3 overlap (ratio of the sham group). The LC3/NLRP3 overlap in the sham group is set to 1. Data are expressed as mean ± SEM (*n* = 5). *P < 0.05 versus sham group. **P < 0.05 versus BCCAo group. ^#^*P* < 0.05 versus BCCAo+URB group

**Fig. 5:**
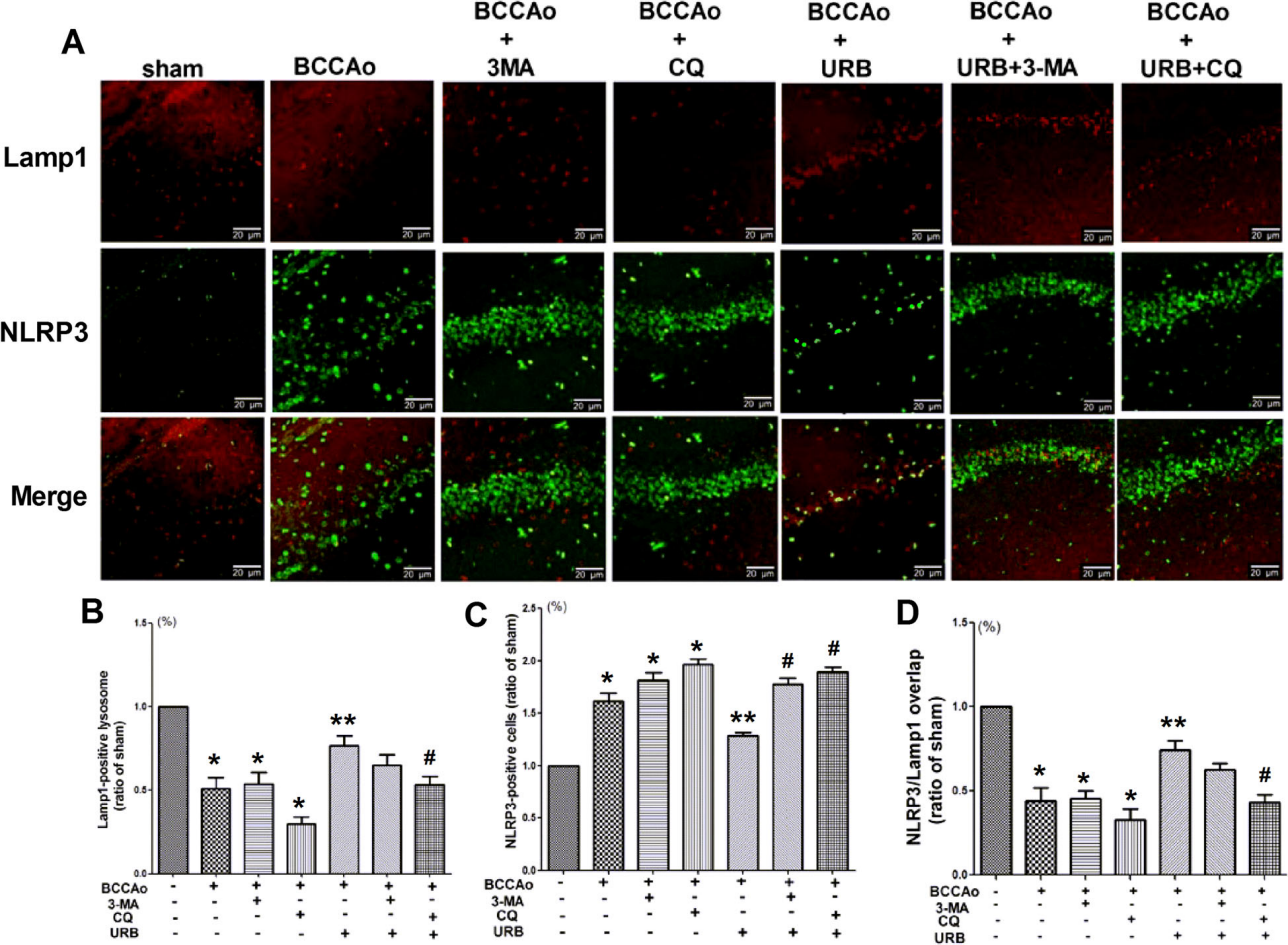
The activation of CCH-induced NLRP3 inflammasome may be restrained by restoring the lysosomal function after URB treatment. **a** Representative immunofluorescence staining for NLRP3 and LAMP1 in the hippocampal CA1 region. (scale bars = 20 μm). *Green puncta*: NLRP3 inflammasome; *red puncta*: LAMP1-positive lysosome; *yellow puncta*: LAMP1/NLRP3 overlap. **b** Relative level of LAMP1 (ratio of the sham group). The LAMP1-positive lysosome in the sham group is set to 1. **c** Relative level of NLRP3 (ratio of the sham group). The NLRP3-positive inflammasome in the sham group is set to 1. **d** Relative level of LAMP1/NLRP3 overlap (ratio of the sham group). The LAMP1/NLRP3 overlap in the sham group is set to 1. Data are expressed as mean ± SEM (*n* = 5). *P < 0.05 versus sham group. **P < 0.05 versus BCCAo group. ^#^*P* < 0.05 versus BCCAo+URB group

Autophagy mainly consists of three sequential steps including autophagosome formation, transportation to the lysosomes and degradation in the lysosomes, and each step may have diverse functions [32]. LC3, p62 and LAMP1 were chosen as the selective markers for the above three steps, respectively. 3-MA, one of the autophagy inhibitors, was used to inhibit autophagosome formation by interrupting PI3K/AKT/mTOR signaling, while CQ, one of the lysosome inhibitors, was utilized to inhibit autophagic flux and lysosomal function by blocking lysosomal acidification [33]. To determine which steps are involved in the effects of URB on CCH-induced NLRP3 inflammasome activation, we examined the expressions of NLRP3, LC3, p62 and LAMP1 and their colocalization in the hippocampus. CCH resulted in the emergence of more NLRP3, LC3 and p62 puncta and fewer LAMP1 puncta, indicating the formation of NLRP3 aggregates, impaired autophagic flux and lysosomal dysfunction, respectively. URB significantly decreased the expressions of NLRP3, LC3 and p62 and increased the expression of LAMP1. Compared with the BCCAo+URB group, the autophagy inhibitor (3-MA+BCCAo+URB group) aggravated the overexpression of NLRP3 and p62 (Figs. 3b, c, 4c, and 5c), while the lysosome inhibitor (CQ+BCCAo+URB group) further increased the expressions of NLRP3, LC3 and p62 and decreased the expression of LAMP1 (Figs. 3b, c, 4b, c, and 5b, c). Confocal microscopy showed very little colocalization between NLRP3 and p62 after URB treatment (Fig. 3a), suggesting that p62 may not be necessary for removing NLRP3 during the process of autophagy after URB treatment. Nevertheless, the fluorescent NLRP3-positive puncta were co-localized with LC3-positive autophagosomes and LAMP1-positive lysosomes respectively, supporting the conclusion that the autophagy-lysosome pathway may involve CCH-induced activation of the NLRP3 inflammasome (Figs. 4a and 5a). Interestingly, URB did not substantially change the overlapping signals of the LC3-stained autophagosomes and NLRP3-positive puncta (Fig. 4d), indicating that the activation of CCH-induced NLRP3 inflammasome may not be restrained by controlling the autophagosome formation stage after URB treatment. However, URB significantly increased the colocalization of NLRP3-positive puncta and LAMP1-positive lysosomes, which was notably reversed by the lysosome inhibitor CQ (Fig. 5d), confirming that URB may suppress the activation of CCH-induced NLRP3 inflammasome by restoring the lysosomal function.

### URB attenuates CCH-induced impaired autophagy by inhibiting microglial overactivation

**Fig. 6:**
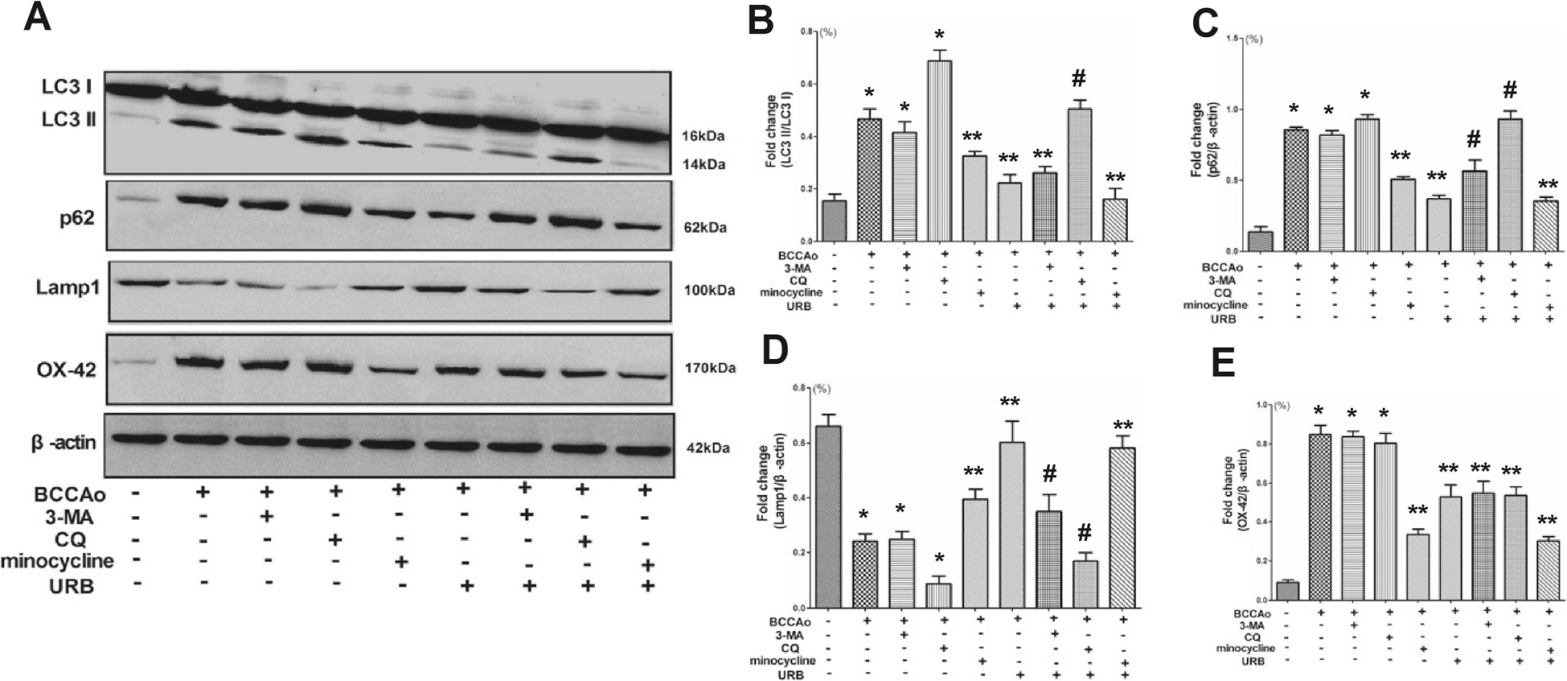
Effects of URB, autophagy inhibitor 3-MA, lysosome inhibitor CQ and microglial activation inhibitor minocycline on CCH-induced impaired autophagy. **a** Representative western blot for LC3, p62, LAMP1, OX-42 and β-actin in the hippocampus. **b** Relative optical density analysis for LC3 in the hippocampus. **c** Relative optical density analysis for p62 in the hippocampus. **d** Relative optical density analysis for LAMP1 in the hippocampus. **e** Relative optical density analysis for OX-42 in the hippocampus. Data are expressed as mean ± SEM (*n* = 5). *P < 0.05 versus sham group. **P < 0.05 versus BCCAo group. ^#^*P* < 0.05 versus BCCAo+URB group

Prior evidence has shown that microglial overactivation may lead to the activation of the NLRP3-CASP1 inflammasome pathway and the release of IL-1β [34–36]. Additionally, autophagy is associated with microglial overactivation in the CNS [37]. Thus, to explore a possible link between abnormal excessive autophagy and microglial overactivation after CCH, we assessed the protein levels of LC3, p62, LAMP1 and OX-42 in the hippocampus. CCH significantly increased the protein levels of LC3, p62 and OX-42 and decreased the protein level of LAMP1, and these effects were reversed by URB treatment (Fig. 6b–e). Intriguingly, the autophagy inhibitor 3-MA and lysosome inhibitor CQ failed to alter the protein level of OX-42 (Fig. 6e), while the microglial activation inhibitor minocycline succeeded in reversing the increases in protein levels of LC3, p62 and LAMP1 induced by CCH (Fig. 6b–d). In addition, URB+minocycline could further restore the protein levels of LC3, p62 and LAMP1 induced by CCH to normal levels (Fig. 6b–e). These data illustrated that microglial overactivation may be the upstream regulator of autophagy after CCH. Moreover, URB may suppress abnormal excessive autophagy induced by CCH in part by inhibiting microglial activation.

### URB inhibits CCH-induced defective mitophagy by preventing ROS accumulation

**Fig. 7:**
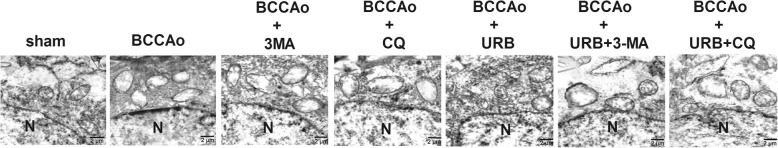
Effects of URB autophagy inhibitor 3-MA and lysosome inhibitor CQ on CCH-induced alterations of mitochondrial ultrastructure. Representative electron micrographs of mitochondria in neurons (scale bars = 2 μm). *N* nucleus

To observe the ultrastructural changes of the mitochondria, brain sections were examined by electron microscopy. In the sham group, we found mitochondria with prominent cristae and an intact membrane. After the induction of CCH, abnormal mitochondrial swelling and vague cristae were observed, which was mitigated by URB treatment. However, the autophagy inhibitor 3-MA and lysosome inhibitor CQ seemed to increase the numbers of these abnormal mitochondria (Fig. 7), indicating that mitophagy may play a critical role in the maintenance of mitochondrial integrity.

**Fig. 8:**
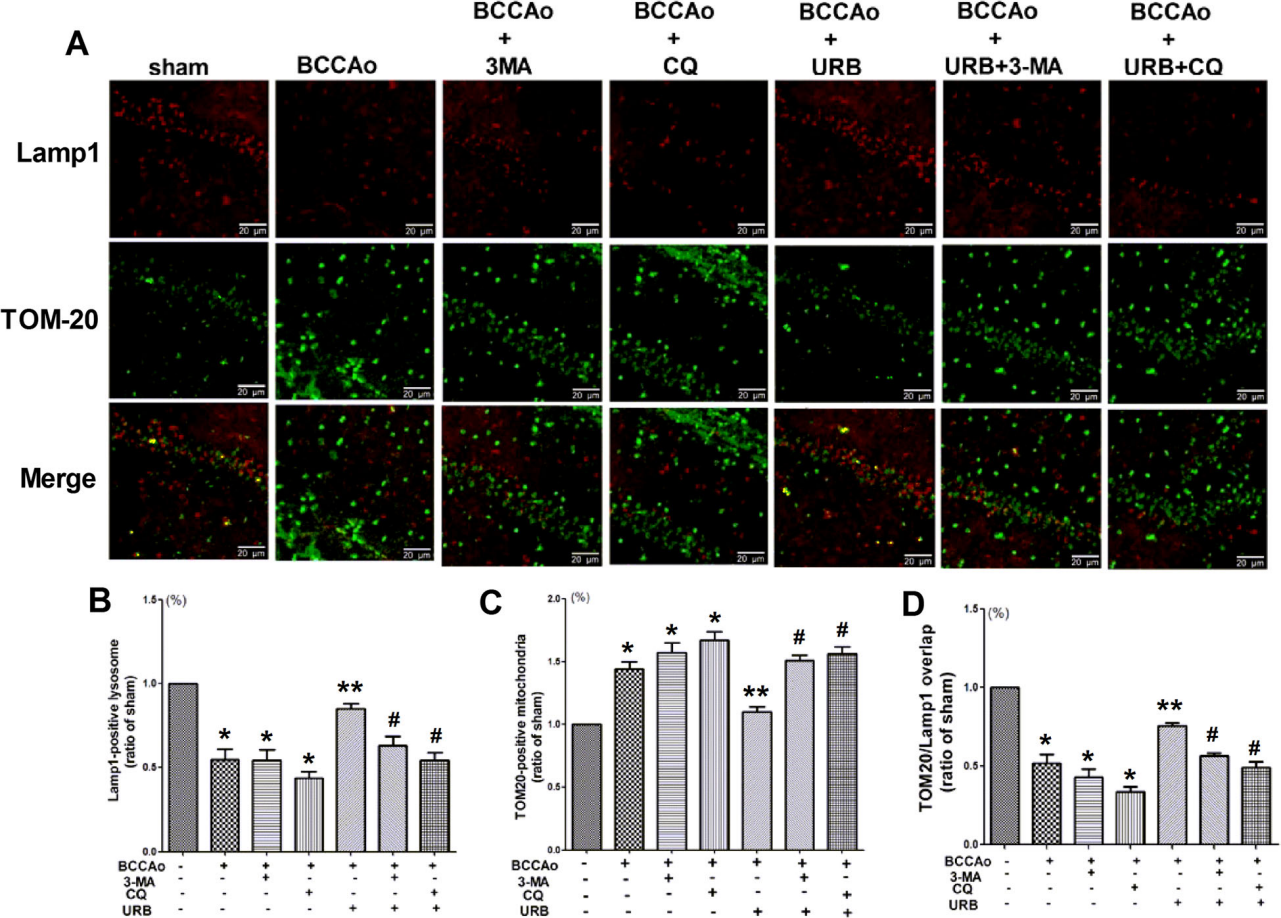
The clearance of CCH-induced damaged mitochondria may be enhanced by restoring the lysosomal function after URB treatment. **a** Representative immunofluorescence staining for LAMP1 and TOM-20 in the hippocampal CA1 region. (scale bars = 20 μm). *Green puncta*: TOM-20-positive mitochondria; *red puncta*: LAMP1-positive lysosome; *yellow puncta*: LAMP1/TOM-20 overlap. **b** Relative level of LAMP1 (ratio of the sham group). The LAMP1-positive lysosome in the sham group is set to 1. **c** Relative level of TOM-20 (ratio of the sham group). The TOM-20-positive mitochondria in the sham group are set to 1. **d** Relative level of LAMP1/TOM-20 overlap (ratio of the sham group). The LAMP1/TOM-20 overlap in the sham group is set to 1. Data are expressed as mean ± SEM (*n* = 5). *P < 0.05 versus sham group. **P < 0.05 versus BCCAo group. ^#^*P* < 0.05 versus BCCAo+URB group

According to the mitophagy pathways [38], damaged mitochondria are transported to the lysosome for degradation via LC3 binding. Thus, we further examined the colocalization of TOM20 and LAMP1 in the hippocampus. Compared with the sham group, the numbers of TOM20-positive mitochondria increased (Fig. 8c), but were rarely co-localized with LAMP1-positive lysosomes after CCH (Fig. 8a). Nevertheless, URB treatment significantly reversed this result, while the lysosome inhibitor CQ+URB further amplified the above phenomenon (Fig. 8a–d), suggesting impaired mitophagy flux.

**Fig. 9:**
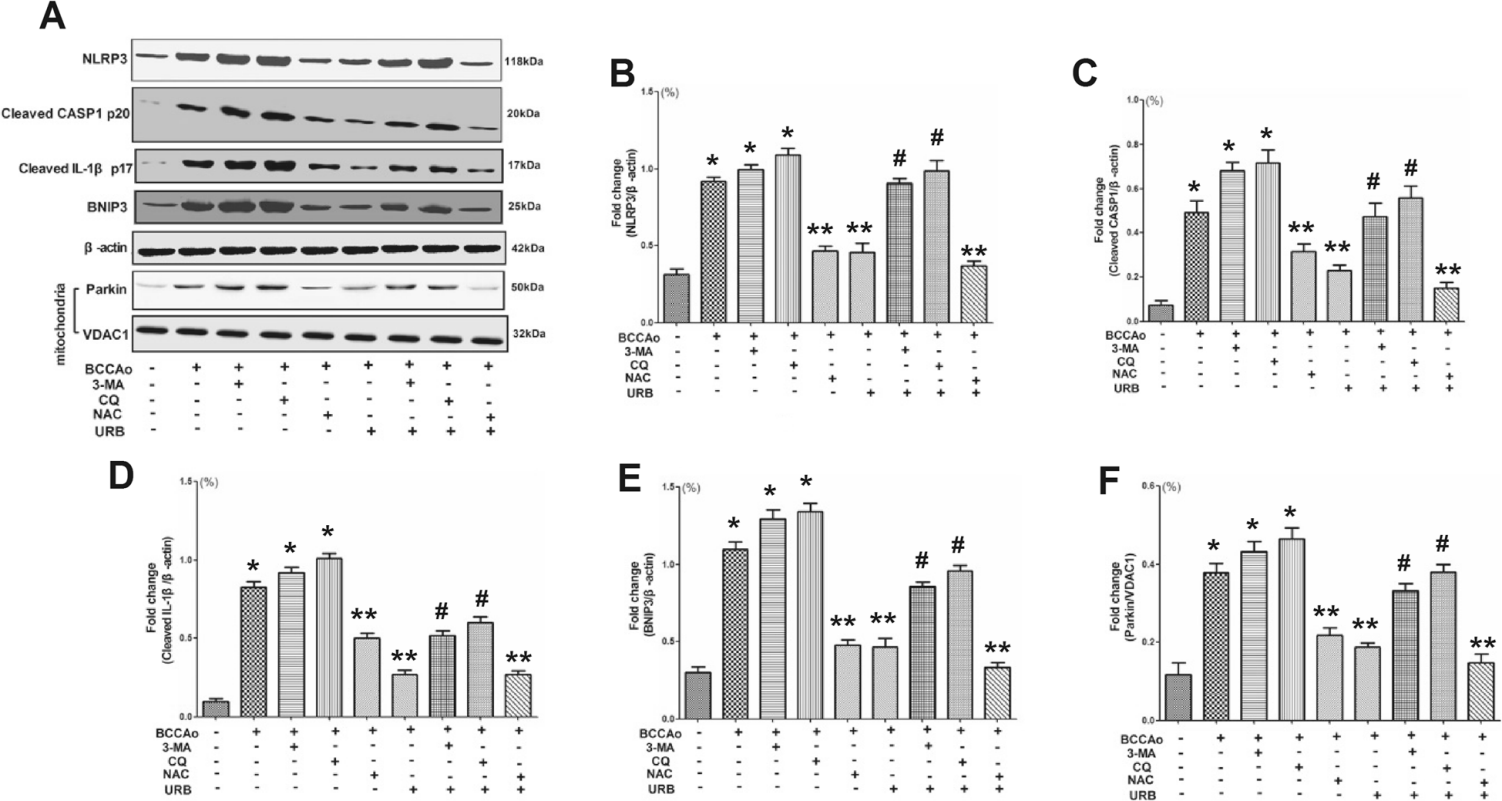
Effects of URB, autophagy inhibitor 3-MA, lysosome inhibitor CQ and ROS scavenger NAC on CCH-induced NLRP3 inflammasome activation and impaired mitophagy. **a** Representative western blot for NLRP3, cleaved CASP1, cleaved IL-1β, BNIP3, parkin, β-actin and VDAC1 in the hippocampus. **b** Relative optical density analysis for NLRP3 in the hippocampus. **c** Relative optical density analysis for cleaved CASP1 in the hippocampus. **d** Relative optical density analysis for cleaved IL-1β in the hippocampus. **e** Relative optical density analysis for BNIP3 in the hippocampus. **f** Relative optical density analysis for parkin in the hippocampus. Data are expressed as mean ± SEM (*n* = 5). *P < 0.05 versus sham group. **P < 0.05 versus BCCAo group. ^#^*P* < 0.05 versus BCCAo+URB group

**Fig. 10:**
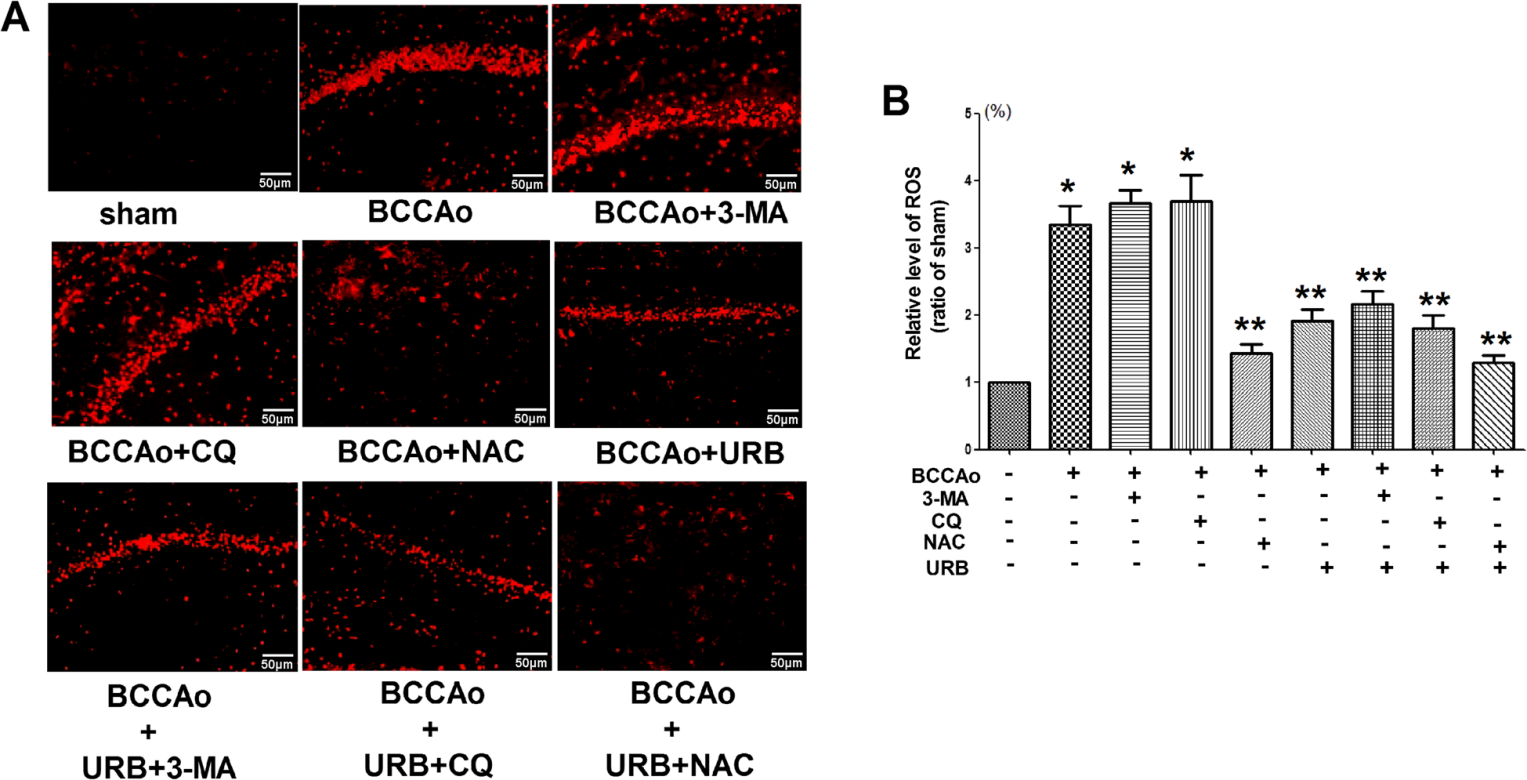
Effects of URB, autophagy inhibitor 3-MA, lysosome inhibitor CQ and ROS scavenger NAC on CCH-induced ROS accumulation. **a** Representative DHE fluorescence staining for ROS in the hippocampal CA1 region (scale bars = 50 μm). **b** Relative level of ROS (ratio of the sham group). The ROS level in the sham group is normalized to 1. Data are expressed as mean ± SEM (*n* = 5). *P < 0.05 versus sham group. **P < 0.05 versus BCCAo group

Mitochondria play a critical role in the activation of the NLRP3 inflammasome [39]. ROS, mainly from the mitochondria [40], may serve as a triggering factor to activate NLRP3 inflammasome [41]. In our previous study [9, 11], we found that CCH led to mitochondrial dysfunction, ROS accumulation and impaired mitophagy. We speculated that impaired mitophagy might be associated with ROS accumulation. Hence, to verify a possible link between impaired mitophagy and ROS accumulation after CCH, the protein levels of two mitophagy markers, parkin and BNIP3 were examined in the hippocampus, and ROS were evaluated by DHE staining. The ROS scavenger *N*-acetylcysteine (NAC) significantly decreased the increased protein levels of NLRP3, cleaved CASP1 and cleaved IL-1β induced by CCH (Fig. 9b–d), thereby confirming ROS accumulation plays a critical role in the activation of the NLRP3 inflammasome after CCH. CCH significantly upregulated the protein levels of parkin and BNIP3 and ROS accumulation, which were markedly downregulated by URB treatment (Figs. 9e, f and 10b). Interestingly, the autophagy inhibitor 3-MA and lysosome inhibitor CQ failed to change the level of ROS (Fig. 10b), whereas the ROS scavenger NAC notably decreased the protein levels of parkin and BNIP3 induced by CCH (Fig. 9e, f). Moreover, URB+NAC could further decrease the protein levels of parkin and BNIP3 induced by CCH to normal levels (Fig. 9e, f). The above results revealed that ROS accumulation may be the upstream triggering factor of mitophagy after CCH. Moreover, URB may mitigate defective mitophagy induced by CCH in part by preventing ROS accumulation.

## Discussion

Owing to the complex mechanisms involved in autophagy and neuroinflammatory responses, the interactions between autophagy and neuroinflammation are controversial. The autophagy proteins function as inducers and suppressors of inflammatory responses, while inflammatory signals could promote or inhibit the process of autophagy [21]. Focusing on chronic cerebral ischemia, in accordance with one recent study [42], we observed the activation of the NLRP3-CASP1 inflammasome pathway and the release of IL-1β after CCH. In addition, we confirmed for the first time, to our knowledge, that lysosomal dysfunction might contribute to the CCH-induced activation of the NLRP3 inflammasome and subsequent release of IL-1β. Microglial overactivation and ROS accumulation may be the upstream triggering factors of autophagy and mitophagy, respectively. Furthermore, we illustrated that URB might inhibit CCH-induced activation of the NLRP3-CASP1 inflammasome pathway through the restoration of lysosomal function, and mitigate the impaired autophagy and mitophagy flux in part by preventing microglial overactivation and ROS accumulation, indicating that URB is a promising therapeutic agent for the treatment of CCH.

We previously demonstrated that CCH impaired cognitive function and neuronal plasticity [8]. IL-1β is a selective proinflammatory cytokine involved in hippocampal-dependent memory consolidation. The accumulation of IL-1β in the hippocampus may contribute to cognitive dysfunction [34]. Maturation and release of IL-1β require the activation of the NLRP3 inflammasome [16]. Consistent with a recent study [2], CCH promoted chronic neuroinflammation through over-expression of NLRP3, cleaved caspase-1 and cleaved IL-1β. The activation of the NLRP3-CASP1 inflammasome pathway in ischemic stroke is based on three canonical hypotheses: ROS accumulation, lysosomal rupture and cellular potassium efflux [19]. At the stage of CCH, ROS accumulation and lysosomal dysfunction-induced impaired autophagy flux contributed to chronic cerebral injury [9, 11]. The above two possible mechanisms regarding the activation of the NLRP3-CASP1 inflammasome pathway after CCH were further investigated. We found the ROS scavenger NAC notably decreased CCH-induced over-expression of NLRP3, cleaved caspase-1 and cleaved IL-1β, indicating that ROS accumulation might play a critical role in the activation of the NLRP3-CASP1 inflammasome signaling after CCH. ROS are mainly generated from mitochondria [39]. Hence, we believe that mitochondrial dysfunction is closely related to the activation of the NLRP3-CASP1 inflammasome pathway. URB treatment could significantly decrease the elevated expression of NLRP3, cleaved caspase-1, cleaved IL-1β and ROS accumulation, suggesting the anti-neuroinflammatory effects of URB. Subsequently, we analyzed the presence, localization and quantity of NLRP3 in the hippocampus and found that it was widely expressed in this region, further confirming the activation of the NLRP3 inflammasome. We observed the colocalization of NLRP3 with LC3-positive autophagosomes and LAMP1-positive lysosomes, indicating that NLRP3 may be transported to the lysosome for degradation via LC3 binding. CCH increased the colocalization of NLRP3 and LC3-positive autophagosomes and decreased the colocalization of NLRP3 and LAMP1-positive lysosomes. In addition, the lysosome inhibitor CQ further increased the overlapping signals of NLRP3 and LC3-stained autophagosomes and decreased the overlapping signals of NLRP3 and LAMP1-stained lysosomes, confirming that lysosomal dysfunction may be responsible for the activation of NLRP3 inflammasome. URB significantly increased the colocalization of NLRP3 and LAMP1-positive lysosomes, which was notably reversed by the lysosome inhibitor CQ, indicating that URB may suppress the activated CCH-induced NLRP3-CASP1 inflammasome by restoring lysosomal function.

P62, the described adaptor protein for autophagic transportation, is involved in the autophagy-mediated inhibition of NLRP3 inflammasome activation [42, 43]. Interestingly, in our present study, p62 was seldom co-localized with NLRP3 after URB treatment, suggesting that p62 may be unnecessary for removing NLRP3 in the process of autophagy after URB treatment. We noticed that p62 is reported to be dispensable for removing damaged mitochondria, challenging the role of p62 in mitophagy [44–46]. Hence, we speculate p62-independent mitophagy might be one of the critical mechanisms involved in NLRP3 inflammasome clearance after URB treatment. In order to eliminate CCH-induced NLRP3 inflammasome activation, other autophagy adaptor proteins, such as NBR1, optineurin, CALCOCO2 and TAX1BP1, might be employed to deliver NLRP3 inflammasome to the lysosome for degradation in p62-independent mitophagy. This possibility requires further investigation.

CCH induced mitochondrial dysfunction, which would promote ROS generation. ROS accumulation may activate the NLRP3-CASP1 inflammasome pathway. According to our electron microscopy results, the autophagy inhibitor 3-MA and lysosome inhibitor CQ further increased the number of CCH-induced abnormal mitochondria, indicating that defective mitophagy may exacerbate the damage to mitochondrial integrity. Subsequently, we confirmed the existence of impaired mitophagy flux after CCH. The mitophagy blockade leads to the accumulation of damaged, ROS-generating mitochondria, which may activate NLRP3-CASP1 inflammasome signaling [47]. Under the condition of CCH, we found both ROS accumulation and mitophagy blockade could promote the activation of NLRP3-CASP1 inflammasome signaling. However, the underlying link between mitophagy and ROS accumulation is not well understood yet. The autophagy inhibitor 3-MA and lysosome inhibitor CQ did not change the level of ROS after CCH, whereas the ROS scavenger NAC notably decreased the protein levels of parkin and BNIP3, indicating that ROS accumulation may be the upstream triggering factor of mitophagy after CCH.

When pathologically insulted, microglia can transform into an “activated” state. Appropriate activation of microglia can be beneficial to neuronal survival, but excessive activation of microglia may aggravate neuronal damage [48]. Evidence has shown that microglial overactivation may lead to the activation of the NLRP3 inflammasome and the release of IL-1β [34–36]. Additionally, the autophagy inhibitor 3-MA and lysosome inhibitor CQ further aggravated CCH-induced increased protein levels of NLRP3, cleaved caspase-1 and cleaved IL-1β, indicating autophagy might participate in CCH-induced activation of the NLRP3 inflammasome and subsequent release of IL-1β. At the stage of CCH, either microglial overactivation or impaired autophagy could lead to the activation of the NLRP3-CASP1 inflammasome pathway. Nevertheless, the possible association between autophagy and microglial overactivation has yet to be clarified. The autophagy inhibitor 3-MA and lysosome inhibitor CQ failed to alter the protein level of OX-42 after CCH, while the microglial activation inhibitor minocycline could reverse the CCH-induced protein levels of LC3, p62 and LAMP1, suggesting that microglial overactivation may be the upstream regulator of autophagy after CCH.

The tight interactions between ROS accumulation and microglial overactivation and autophagy are mainly reflected in two ways: the induction of autophagy by ROS accumulation or microglial overactivation [49–51] and the reduction of ROS accumulation or microglial overactivation via autophagy [52, 53], which is complex, and depends on the region, severity and phase of the insult. In our present study, CCH might impair the processes of autophagy and mitophagy through upregulation of ROS generation and microglial overactivation, indicating that neuroinflammation may play a more dominant role than impaired autophagy at the stage of chronic cerebral ischemic injury. In our opinion, in the presence of an acute ischemic insult, enhanced autophagy could eliminate damaged neuronal cells. Thus, at the initial stage of neuroinflammatory injury, autophagy might regulate the neuroinflammation. With an extension of the insult time and the emergence of CCH-induced autophagy dysfunction, inflammatory neuronal damage gradually cannot be prevented, increasing the accumulation of damaged neuronal cells, and could further exacerbate neuroinflammation, which may aggravate impaired autophagy in turn. Hence, at the stage of chronic neuroinflammation, neuroinflammation may be the upstream regulator of autophagy.

## Conclusions

In conclusion, our data revealed that CCH-induced microglial overactivation and ROS accumulation promoted the activation of the NLRP3 inflammasome and the release of IL-1β. In addition, blocked autophagy and mitophagy flux may contribute to the activation of the NLRP3-CASP1 inflammasome pathway. However, URB might alleviate impaired autophagy and mitophagy by decreasing mitochondrial ROS and microglial overactivation as well as restoring lysosomal function, which would further inhibit the activation of the NLRP3-CASP1 inflammasome pathway. These findings extend and underscore the potential therapeutic effects of URB in chronic cerebral ischemic insults.

## Data Availability

The data used in this article are available to researchers subject to confidentiality if necessary.

## References

[1] Arsava EM, Hansen MB, Kaplan B, Peker A, Gocmen R, Arat A, Oguz KK, Topcuoglu MA, Østergaard L, Dalkara T (2018). The effect of carotid artery stenting on capillary transit time heterogeneity in patients with carotid artery stenosis. Eur Stroke J.

[2] Shang J, Yamashita T, Zhai Y, Nakano Y, Morihara R, Li X, Tian F, Liu X, Huang Y, Shi X, Sato K, Takemoto M, Hishikawa N, Ohta Y, Abe K (2019). Acceleration of NLRP3 inflammasome by chronic cerebral hypoperfusion in Alzheimer’s disease model mouse. Neurosci Res.

[3] Hai J, Su SH, Lin Q, Zhang L, Wan JF, Li H, Chen YY, Lu Y (2010). Cognitive impairment and changes of neuronal plasticity in rats of chronic cerebral hypoperfusion associated with cerebral arteriovenous malformations. Acta Neurol Belg.

[4] Chen L, Mao Y, Zhou LF (2009). Local chronic hypoperfusion secondary to sinus high pressure seems to be mainly responsible for the formation of intracranial dural arteriovenous fistula. Neurosurgery.

[5] Choi SA, Chong S, Kwak PA, Moon YJ, Jangra A, Phi JH, Lee JY, Park SH, Kim SK (2018). Impaired functional recovery of endothelial colony-forming cells from moyamoya disease in a chronic cerebral hypoperfusion rat model. J Neurosurg Pediatr.

[6] Hainsworth AH, Markus HS (2008). Do in vivo experimental models reflect human cerebral small vessel disease? A systematic review. J Cereb Blood Flow Metab.

[7] Su SH, Wu YF, Lin Q, Hai J (2015). Cannabinoid receptor agonist WIN55,212-2 and fatty acid amide hydrolase inhibitor URB597 suppress chronic cerebral hypoperfusion-induced neuronal apoptosis by inhibiting c-Jun N-terminal kinase signaling. Neuroscience.

[8] Su SH, Wang YQ, Wu YF, Lin Q, Hai J (2016). Cannabinoid receptor agonist WIN55,212-2 and fatty acid amide hydrolase inhibitor URB597 may protect against cognitive impairment in rats of chronic cerebral hypoperfusion via PI3K/AKT signaling. Behav Brain Res.

[9] Su SH, Wu YF, Lin Q, Hai J (2017). Cannabinoid receptor agonist WIN55,212-2 and fatty acid amide hydrolase inhibitor URB597 ameliorate neuroinflammatory responses in chronic cerebral hypoperfusion model by blocking NF-κB pathways. Naunyn Schmiedeberg’s Arch Pharmacol.

[10] Wang D, Lin Q, Su S, Liu K, Wu Y, Hai J (2017). URB597 improves cognitive impairment induced by chronic cerebral hypoperfusion by inhibiting mTOR-dependent autophagy. Neuroscience.

[11] Su SH, Wu YF, Wang DP, Hai J (2018). Inhibition of excessive autophagy and mitophagy mediates neuroprotective effects of URB597 against chronic cerebral hypoperfusion. Cell Death Dis.

[12] Bond WS, Rex TS (2014). Evidence that erythropoietin modulates neuroinflammation through differential action on neurons, astrocytes, and microglia. Front Immunol.

[13] Hovens IB, van Leeuwen BL, Nyakas C, Heineman E, van der Zee EA, Schoemaker RG (2015). Postoperative cognitive dysfunction and microglial activation in associated brain regions in old rats. Neurobiol Learn Mem.

[14] Lozano D, Gonzales-Portillo GS, Acosta S, de la Pena I, Tajiri N, Kaneko Y, Borlongan CV (2015). Neuroinflammatory responses to traumatic brain injury: etiology, clinical consequences, and therapeutic opportunities. Neuropsychiatr Dis Treat.

[15] Kanneganti TD, Lamkanfi M, Núñez G (2007). Intracellular NOD-like receptors in host defense and disease. Immunity.

[16] Schroder K, Tschopp J (2010). The inflammasomes. Cell.

[17] Davis BK, Wen H, Ting JP (2011). The inflammasome NLRs in immunity, inflammation, and associated diseases. Annu Rev Immunol.

[18] Pan Y, Chen XY, Zhang QY, Kong LD (2014). Microglial NLRP3 inflammasome activation mediates IL-1β-related inflammation in prefrontalcortex of depressive rats. Brain Behav Immun.

[19] Hong P, Gu RN, Li FX, Xiong XX, Liang WB, You ZJ, Zhang HF (2019). NLRP3 inflammasome as a potential treatment in ischemic stroke concomitant with diabetes. J Neuroinflammation.

[20] Choi AM, Ryter SW, Levine B (2013). Autophagy in human health and disease. N Engl J Med.

[21] Su P, Zhang J, Wang D, Zhao F, Cao Z, Aschner M, Luo W (2016). The role of autophagy in modulation of neuroinflammation in microglia. Neuroscience..

[22] Farkas E, Luiten PG, Bari F (2017). Permanent, bilateral common carotid artery occlusion in the rat: a model for chronic cerebral hypoperfusion-related neurodegenerative diseases. Brain Res Rev.

[23] Ashraf T, Jiang W, Hoque MT, Henderson J, Wu C, Bendayan R (2014). Role of anti-inflammatory compounds in human immunodeficiency virus-1 glycoprotein120-mediated brain inflammation. J Neuroinflammation.

[24] Yew WP, Djukic ND, Jayaseelan JSP, Walker FR, Roos KAA, Chataway TK, Muyderman H, Sims NR (2019). Early treatment with minocycline following stroke in rats improves functional recovery and differentially modifies responses of peri-infarct microglia and astrocytes. J Neuroinflammation.

[25] Di-Pietro PB, Dias ML, Scaini G, Burigo M, Constantino L, Machado RA, Dal-Pizzol F, Streck EL (2008). Inhibition of brain creatine kinase activity after renal ischemia is attenuated by N-acetylcysteine and deferoxamine administration. Neurosci Lett.

[26] Paxinos G, Franklin K (2001). The mouse brain in stereotaxic coordinates.

[27] Lu J, Wu DM, Zheng YL, Hu B, Zhang ZF (2010). Purple sweet potato color alleviates D-galactose-induced brain aging in old mice by promoting survival of neurons via PI3K pathway and inhibiting cytochrome C-mediated apoptosis. Brain Pathol.

[28] Poulet R, Gentile MT, Vecchione C, Distaso M, Aretini A, Fratta L, Russo G, Echart C, Maffei A, De Simoni MG, Lembo G (2005). Acute hypertension induces oxidative stress in brain tissues. J Cereb Blood Flow Metab.

[29] Han X, Sun S, Sun Y, Song Q, Zhu J, Song N, Chen M, Sun T, Xia M, Ding J, Lu M, Yao H, Hu G. Small molecule-driven NLRP3 inflammation inhibition via interplay between ubiquitination and autophagy: implications for Parkinson disease. Autophagy. 2019:1–22. 10.1080/15548627.2019.1596481 PMC6844502 30966861

[30] Shi CS, Shenderov K, Huang NN, Kabat J, Abu-Asab M, Fitzgerald KA, Sher A, Kehrl JH (2012). Activation of autophagy by inflammatory signals limits IL-1β production by targeting ubiquitinated inflammasomes for destruction. Nat Immunol.

[31] Heneka MT, Kummer MP, Stutz A, Delekate A, Schwartz S, Vieira-Saecker A, Griep A, Axt D, Remus A, Tzeng TC, Gelpi E, Halle A, Korte M, Latz E, Golenbock DT (2013). NLRP3 is activated in Alzheimer’s disease and contributes to pathology in APP/PS1 mice. Nature.

[32] Mizushima N (2007). Autophagy: process and function. Genes Dev.

[33] Lee JH, Rao MV, Yang DS, Stavrides P, Im E, Pensalfini A, Huo C, Sarkar P, Yoshimori T, Nixon RA (2019). Transgenic expression of a ratiometric autophagy probe specifically in neurons enables the interrogation of brain autophagy in vivo. Autophagy.

[34] Wang D, Zhang J, Jiang W, Cao Z, Zhao F, Cai T, Aschner M, Luo W (2017). The role of NLRP3-CASP1 in inflammasome mediated neuroinflammation and autophagy dysfunction in manganese-induced, hippocampal dependent impairment of learning and memory ability. Autophagy.

[35] Liu X, Quan N (2018). Microglia and CNS interleukin-1: beyond immunological concepts. Front Neurol.

[36] He W, Long T, Pan Q, Zhang S, Zhang Y, Zhang D, Qin G, Chen L, Zhou J (2019). Microglial NLRP3 inflammasome activation mediates IL-1β release and contributes to centralsensitization in a recurrent nitroglycerin-induced migraine model. J Neuroinflammation.

[37] Arroyo DS, Gaviglio EA, Peralta Ramos JM, Bussi C, Rodriguez-Galan MC, Iribarren P (2014). Autophagy in inflammation, infection, neurodegeneration and cancer. Int Immunopharmacol.

[38] Wang Y, Liu N, Lu B (2019). Mechanisms and roles of mitophagy in neurodegenerative diseases. CNS Neurosci Ther.

[39] Iyer SS, He Q, Janczy JR, Elliott EI, Zhong Z, Olivier AK, Sadler JJ, Knepper-Adrian V, Han R, Qiao L, Eisenbarth SC, Nauseef WM, Cassel SL, Sutterwala FS (2013). Mitochondrial cardiolipin is required for Nlrp3 inflammasome activation. Immunity.

[40] Leemans JC, Cassel SL, Sutterwala FS (2011). Sensing damage by the NLRP3 inflammasome. Immunol Rev.

[41] Abais JM, Xia M, Zhang Y, Boini KM, Li PL (2015). Redox regulation of NLRP3 inflammasomes: ROS as trigger or effector?. Antioxid Redox Signal.

[42] Liu C, Wang J, Yang Y, Liu X, Zhu Y, Zou J, Peng S, Le TH, Chen Y, Zhao S, He B, Mi Q, Zhang X, Du Q (2018). Ginsenoside Rd ameliorates colitis by inducing p62-driven mitophagy-mediated NLRP3 inflammasome inactivation in mice. Biochem Pharmacol.

[43] Zhong Z, Umemura A, Sanchez-Lopez E, Liang S, Shalapour S, Wong J, He F, Boassa D, Perkins G, Ali SR, McGeough MD, Ellisman MH, Seki E, Gustafsson AB, Hoffman HM, Diaz-Meco MT, Moscat J, Karin M (2016). NF-KB restricts inflammasome activation via elimination of damaged mitochondria. Cell.

[44] Narendra D, Kane LA, Hauser DN, Fearnley IM, Youle RJ (2010). p62/SQSTM1 is required for Parkin-induced mitochondrial clustering but not mitophagy; VDAC1 is dispensable for both. Autophagy.

[45] Strappazzon F, Nazio F, Corrado M, Cianfanelli V, Romagnoli A, Fimia GM, Campello S, Nardacci R, Piacentini M, Campanella M, Cecconi F (2015). AMBRA1 is able to induce mitophagy via LC3 binding, regardless of PARKIN and p62/SQSTM1. Cell Death Differ.

[46] Houtman J, Freitag K, Gimber N, Schmoranzer J, Heppner FL, Jendrach M (2019). Beclin1-driven autophagy modulates the inflammatory response of microglia via NLRP3. EMBO J.

[47] Zhou R, Yazdi AS, Menu P, Tschopp J (2011). A role for mitochondria in NLRP3 inflammasome activation. Nature.

[48] Fleming JC, Norenberg MD, Ramsay DA, Dekaban GA, Marcillo AE, Saenz AD, Pasquale-Styles M, Dietrich WD, Weaver LC (2006). The cellular inflammatory response in human spinal cords after injury. Brain.

[49] Du D, Hu L, Wu J, Wu Q, Cheng W, Guo Y, Guan R, Wang Y, Chen X, Yan X, Zhu D, Wang J, Zhang S, Guo Y, Xia C (2017). Neuroinflammation contributes to autophagy flux blockage in the neurons of rostral ventrolateral medulla in stress-induced hypertension rats. J Neuroinflammation.

[50] Jassey A, Liu CH, Changou CA, Richardson CD, Hsu HY, Lin LT (2019). Hepatitis C virus non-structural protein 5A (NS5A) disrupts mitochondrial dynamics and induces mitophagy. Cells.

[51] Thangaraj A, Periyasamy P, Guo ML, Chivero ET, Callen S, Buch S. Mitigation of cocaine-mediated mitochondrial damage, defective mitophagy and microglial activation by superoxide dismutase mimetics. Autophagy. 2019:1–24. 10.1080/15548627.2019.1607686 PMC6984592 30990365

[52] Zhang F, Dong H, Lv T, Jin K, Jin Y, Zhang X, Jiang J (2018). Moderate hypothermia inhibits microglial activation after traumatic brain injury by modulating autophagy/apoptosis and the MyD88-dependent TLR4 signaling pathway. J Neuroinflammation.

[53] Ureshino RP, Rocha KK, Lopes GS, Trindade CB, Smaili SS (2014). Calcium signaling alterations, oxidative stress and autophagy in aging. Antioxid Redox Signal.

